# The Impact of Stenosis Severity on Hemodynamic Parameters in the Iliac Artery: A Fluid–Structure Interaction Study

**DOI:** 10.3390/bioengineering12101042

**Published:** 2025-09-28

**Authors:** Nima Rahmati, Hamidreza Pouraliakbar, Arshia Eskandari, Kian Javari, Alireza Jabbarinick, Parham Sadeghipour, Madjid Soltani, Mona Alimohammadi

**Affiliations:** 1 Department of Mechanical Engineering, K. N. Toosi University of Technology, Tehran 19991-43344, Iran; n_rahmati@yahoo.com (N.R.); arshiaeskandari.bio@gmail.com (A.E.); kjavari@gmail.com (K.J.); alirezajabbarinick7158@gmail.com (A.J.); 2 Cardiovascular Imaging Department, Rajaie Cardiovascular Medical and Research Center, Iran University of Medical Sciences, Tehran 19956-14331, Iran; hamidpou@yahoo.com; 3 Vascular Disease and Thrombosis Research Center, Rajaie Cardiovascular Institute, Tehran 19956-14331, Iran; 4 Department of Electrical and Computer Engineering, University of Waterloo, Waterloo, ON N2L 3G1, Canada; 5 Centre for Sustainable Business, International Business University, Toronto, ON M5S 2V1, Canada; 6 Balsillie School of International Affairs (BSIA), Waterloo, ON N2L 6C2, Canada; 7 Centre for Biotechnology and Bioengineering (CBB), University of Waterloo, Waterloo, ON N2L 3G1, Canada; 8 Department of Integrative Oncology, BC Cancer Research Institute, Vancouver, BC V5Z 1L3, Canada

**Keywords:** hemodynamics, iliac artery, fluid–structural interaction, windkessel model, stenosis severity, semi-idealized geometries

## Abstract

The common iliac artery supplies blood to the lower extremities, and stenosis in this region severely impacts hemodynamics. This study investigates the effects of 25%, 50%, and 75% iliac artery stenosis on key hemodynamic parameters using a fluid–structure interaction (FSI) approach. Semi-idealized geometries reconstructed from patient-specific data modeled realistic arterial behavior. Parameters such as wall displacement, time-averaged wall shear stress (TAWSS), oscillatory shear index (OSI), high oscillatory low shear magnitude (HOLMES) index, and endothelial cell activation potential (ECAP) were evaluated. Results showed peak wall displacement of 2.85 mm in the bifurcation zone under 75% stenosis. TAWSS increased with stenosis severity, peaking in stenotic regions and decreasing significantly downstream. OSI was highest in non-stenosed right branches and bifurcation areas, indicating multidirectional shear forces. HOLMES values were lowest downstream of stenoses, indicating disturbed flow. ECAP exceeded the thrombosis risk threshold (1.4 Pa^−1^) in post-stenotic zones under 75% stenosis, suggesting a higher risk of clot formation. These results demonstrate that stenosis disrupts local flow and causes hemodynamic changes downstream, emphasizing the need for comprehensive clinical assessment beyond the stenotic site. Regions with elevated ECAP and low HOLMES downstream may be prone to thrombosis, highlighting the importance of careful hemodynamic monitoring for treatment strategies.

## 1. Introduction

Cardiovascular diseases (CVDs) are the most prevalent diseases worldwide, accounting for a significant portion of global mortality [1]. At the end of 2023, over 500 million individuals worldwide were suffering from CVDs [2]. Despite advancements in science and technology, CVDs continue to claim numerous lives, with 20.5 million deaths attributed to CVDs in 2021 [2]. This represents one-third of total deaths in that year and marks an increase of approximately 8 million deaths compared to 1990 [2]. CVDs encompass a diverse spectrum of conditions affecting the heart and blood vessels. These include aortic diseases (i.e., aneurysms or dissections), peripheral arterial diseases, valvular heart disease, inherited heart defects, hypertension, arrhythmias, and more [1]. Each of these conditions has the potential to lead to severe complications, including cardiac arrest, stroke, heart failure, and ultimately, death [3]. The common iliac artery, a critical vessel supplying blood to the lower limbs, is susceptible to these diseases and their consequences, just like any other vital artery in the human body [4]. Among CVDs affecting the iliac artery, stenosis and plaque burden (atherosclerosis) are particularly prevalent [5]. Arterial stenosis is a serious condition requiring prompt medical attention [6]. Early detection may allow for management with medication; however, severe stenosis necessitates immediate intervention, potentially including stenting or even open-heart surgery [7]. The choice of treatment depends on the severity of the stenosis. Typically, patients with critical stenosis and functional limitations (i.e., claudication) or patients with critical limb ischemia (CLI) need medical attention [8]. Therefore, they could potentially be candidates for open surgical repair or endovascular treatment [9].

Numerical modeling and simulations offer valuable tools for investigating blood flow within arteries and determine whether there is a need for follow up or not [10]. These simulations can be used to analyze hemodynamic parameters, which are essential for understanding blood flow dynamics [11]. While prior computational studies of the iliac artery have provided valuable insights, a distinct research gap remains. For instance, Alimohammadi et al. employed Computational Fluid Dynamics (CFD) for plaque deposition prediction in iliac bifurcations affected by aortic dissection but used rigid wall assumptions, limiting accuracy in stenotic regions where wall compliance becomes critical [12]. Carvalho et al. investigated unsteady flow in iliac bifurcations but similarly neglected fluid–structure interaction (FSI) and non-Newtonian blood behavior [13]. Carneiro et al. and Harris et al. modeled blood as Newtonian fluid with constant viscosity, failing to capture shear-thinning behavior that significantly affects flow patterns in stenotic regions [14,15]. These studies also assumed rigid arterial walls, neglecting the crucial fluid–structure interaction.

Although some FSI studies exist, there has been no systematic analysis demonstrating how a progression of stenosis severity (from mild to severe) impacts a comprehensive suite of advanced hemodynamic parameters, particularly those linked to thrombosis risk. For instance, Luo et al. performed FSI analysis on iliac bifurcation but did not systematically investigate stenosis severity effects or incorporate comprehensive hemodynamic parameter analysis [16]. Skopalik et al. and Heinen et al. focused primarily on pressure gradient estimation rather than comprehensive hemodynamic characterization and did not combine FSI with non-Newtonian blood modeling [17,18,19].

Therefore, this study aims to address these gaps by integrating three critical components: first, patient-derived geometry with FSI to capture wall mechanics; second, a non-Newtonian blood model for physiological accuracy, and third, a multi-stage stenosis analysis with comprehensive hemodynamic parameter evaluation to map the progressive degradation of hemodynamic health. This approach provides a more complete picture of the downstream consequences of iliac stenosis, which has significant clinical implications.

In addition, this study aims to utilize synthetic geometries reconstructed from patient-specific data to improve the model’s applicability while maintaining computational efficiency. It also investigates the impact of varying degrees of stenosis on hemodynamic parameters within the iliac artery. The findings are intended to advance the understanding of how stenosis severity influences blood flow behavior in patient-specific iliac artery bifurcations. Such insights may support the development of more accurate diagnostic tools and targeted treatment strategies for iliac artery disease.

## 2. Materials and Methods

### 2.1. Data Population

In this study, CT scan images of a patient were used for three-dimensional reconstruction and geometry generation. The imaging data from an 18-year-old male patient with a healthy iliac artery was used, and then changes were made to create stenosis in it. The study was performed under IR.IUMS.FMD.REC.1401.257 ethical number. The study protocol was approved by the Rajaie Cardiovascular Medical and Research Center ethics committee and the participant signed informed consent, and all methods were performed according to the relevant guidelines and regulations.

### 2.2. Geometry Reconstruction

To extract the three-dimensional geometry, CT scan images in DICOM format were imported into MIMICS Research 21.0 (Materialise, Leuven, Belgium), and the geometry was reconstructed in STL format. To investigate different degrees of stenosis, the reconstructed geometry was modified to create three cases with asymmetric stenoses of 25%, 50%, and 75% severity in the left common iliac branch, reflecting the prevalence of stenosis formation in this region [20]. The left common iliac artery was chosen as the location for the stenosis because it is a common site for atherosclerotic plaque development, partly due to the anatomical compression by the overlying right common iliac artery (a condition related to May–Thurner syndrome) [20]. The severities of 25%, 50%, and 75% were chosen to represent mild, moderate, and severe levels of disease, which correspond to different stages in clinical assessment and treatment decision-making [8,21].

The stenoses were created in Free CAD 1.0 software by defining a focal region on one side of the left common iliac artery wall. A smooth, cosine-shaped lesion was then generated, reducing the local cross-sectional area by 25%, 50%, and 75% relative to the original, healthy vessel area. This method created an asymmetric stenosis, which is more representative of typical atherosclerotic plaque growth [22].

All three cases in this study are semi-idealized geometries. Here, semi-idealized geometries means that the primary anatomical features (such as the bifurcation angle, the diameters of the common, internal, and external iliac, and the overall curvature) were preserved from the patient-specific data. The idealization involved smoothing the vessel surface to remove minor imaging artifacts and creating geometrically controlled, well-defined stenoses for systematic comparison.

### 2.3. Mesh Generation

**Figure 1:**
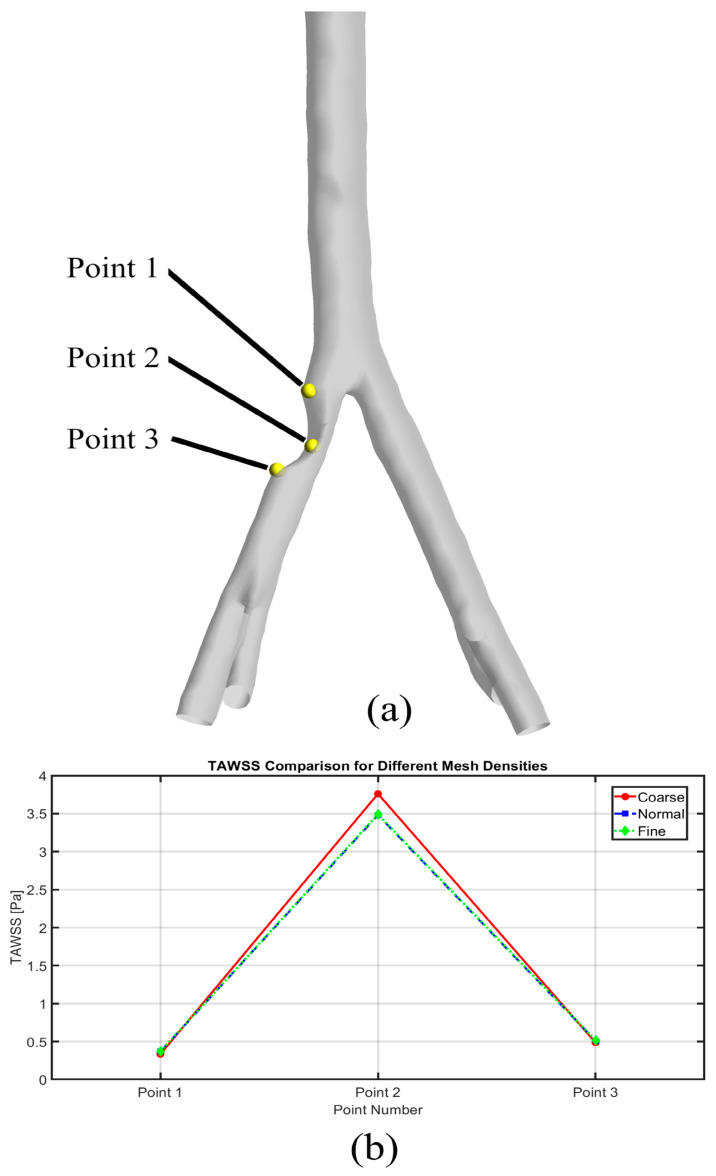
Mesh sensitivity analysis: (**a**): three different points to calculate time-averaged wall shear stress values; (**b**): time-averaged wall shear stress values in three different sets of meshes.

Meshing of all geometries was performed using the ANSYS Meshing 2019 R3 (ANSYS Inc., Canonsburg, PA, USA) software. To better simulate the boundary layers around the fluid wall, the inflation layer and the first layer thickness method were used. For the mesh sensitivity analysis, three sets of mesh elements were used for all cases, and eventually to compromise for both accuracy and computational time, the medium meshes with approximately 315,000 and 65,000 elements were chosen for fluid and solid domains, respectively. Figure 1 demonstrates time-averaged wall shear stress values in three sets of meshes (coarse, medium, and fine) in three different points. The figure shows the overlap between medium and fine mesh.

### 2.4. Computational Analysis

The continuity equation (Equation (1)) and the Cauchy momentum equations (Equation (2)) were used to solve the fluid flow behavior in this study.

(1) ∇⋅U=0

(2) $$\rho \frac{Dv}{Dt}=\rho f+\nabla$$

**Figure 2:**
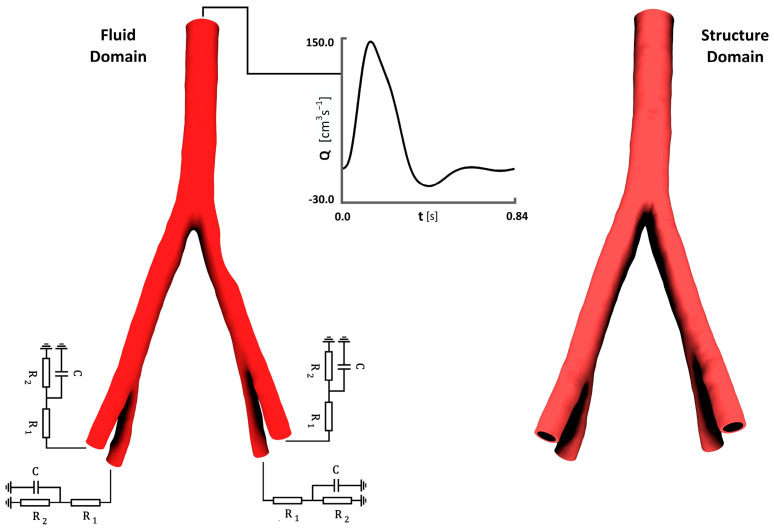
Flow wave for inlet boundary condition and Windkessel model for outlet.

In this study, the flow rate curve presented in previous articles was used for the inlet boundary condition [23]. Figure 2 shows the flow rate–time curve that was used as the boundary condition for different degrees of stenosis. It also illustrates the schematic of the placement of RCR circuits at the four outlets of the geometry under consideration.

Flow-dependent pressure was used for the outlet boundary condition in each simulation. To determine the pressure as the outlet boundary condition, the 3-element Windkessel zero-dimensional (0D) model was used. This model simulates the pressure after the outlet as an electrical circuit in which current and pressure play a key role. The 3-element Windkessel model used in this study consists of two resistances (R1, R2) and one compliance (C). The 3-element Windkessel model is given in the Equation (3) and the process of deriving and tuning Windkessel parameters for each outlet was based on the work of Alimohammadi et al. [24]. Hence, the parameters (R1, R2, C) for each of the four outlets were tuned based on physiological principles and to match the characteristics of the downstream vasculature, following the methodology and pressure data detailed in the work of Alimohammadi et al. [24]. This ensures that the pressure waves are correctly propagated and reflected at the outlets, mimicking the behavior of the distal circulatory system. The specific resistance and compliance values used for the right internal iliac artery (RIIA), right external iliac artery (REIA), left internal iliac artery (LIIA), and left external iliac artery (LEIA) outlets are detailed in Table 1.

(3) $$P=Q\left(R_{1}+R_{2}\right)-{\;R}_{2}C\frac{dP}{dt}+R_{1}R_{2}C\frac{dQ}{dt}$$

Blood is considered a non-Newtonian fluid with shear-thinning behavior. Specifically, when studying smaller arteries, it is crucial to account for the non-Newtonian properties of blood to ensure modeling accuracy [25]. In this study, blood is modeled as an incompressible, non-Newtonian fluid with a density of 1060 kg/m^3^ [26]. The Carreau–Yasuda model is employed to simulate the non-Newtonian behavior of blood due to its high accuracy in representing shear-thinning effects. The model is presented in Equation (4), and the parameters used were obtained from the work of Gijsen et al. [27]. Table 2 shows the parameters that were used for the Carreau–Yasuda model in this study.

In this equation, μ represents viscosity, μ_0_ represents low shear viscosity, μ_∞_ represents high shear viscosity, λ_CY_ acts as a time constant, γ′ represents shear rate, m is the Yasuda power law exponent, and a is the power law index.

(4) $$\mu =\left(\mu_{0}-\mu_{\infty }\right){\left(1+{\left(\lambda_{CY}\gamma^{\prime }\right)}^{a}\right)}^{(m-1)/a}+\mu_{\infty }$$

For the analysis of the arterial wall, the vessel was modeled as a homogeneous, isotropic, and linear elastic material with a uniform thickness of 1.4 mm. Furthermore, the wall density was set to 1120 Kg/m^3^. The linear elastic model was utilized, with a Young’s modulus of 2 Mpa and a Poisson’s ratio of 0.4, as reported in the study by Skacel et al. and Camasão et al., respectively [28,29].

The interaction between the blood flow and the deformable arterial wall was simulated using a robust two-way FSI coupling approach, implemented within the ANSYS 2019 R3 (ANSYS Inc., Canonsburg, PA, USA) software environment. The simulation employed an iterative, partitioned procedure where the fluid and solid domains were solved sequentially at each time step. This two-way data exchange continued until convergence was achieved for each time step, ensuring that the dynamic interplay between fluid forces and structural deformation was fully captured throughout the cardiac cycle. Simulations were transient and the time step was set to Δt = 0.001 (s), and we simulated three cardiac cycles to ensure convergence.

### 2.5. Hemodynamic Parameters

Velocity, pressure, wall displacement, time-averaged wall shear stress (TAWSS), oscillatory shear index (OSI), high oscillatory low shear magnitude (HOLMES), and endothelial cell action potential (ECAP) are examined as important parameters that contribute to a better understanding of the simulation results.

#### 2.5.1. TAWSS

Wall shear stress (WSS) is one of the most important parameters that must be considered in cardiovascular simulations. This is because WSS has a significant impact on the structure of the inner wall of blood vessels. The mean magnitude of shear stress that is applied to the wall over time is called the TAWSS [30]. Low and high values of TAWSS can cause damage to the vessel wall, a topic that has been the subject of much discussion in the engineering and medical community [31]. Therefore, examining TAWSS in cardiovascular simulations can provide valuable and unique insights. Equation (5) represents the TAWSS. In this equation, T is the duration of the cardiac cycle, and the absolute value of wall shear stress represents the magnitude of wall shear stress.

(5) $$TAWSS=\frac{1}{T}\left(\int_{0}^{T}\left|\tau_{w}\right|dt\right)\;$$

#### 2.5.2. OSI

OSI is an index that indicates the magnitude of the oscillatory forces that are applied to the inner wall of the vessel and endothelial cells [32]. OSI represents the direction of shear stress applied to the wall. The value of this index is typically in 0 and 0.5 intervals. When this index is 0, it means that the flow is unidirectional, and the stress on the wall is applied in only one direction. When this value approaches 0.5, it means that there is no specific direction for the flow, and the shear stress is applied to the wall in an oscillatory manner and in different directions. Equation (6) represents the OSI:

(6) $$OSI\;=\frac{1}{2}\left(\frac{1\;-\frac{1}{T}\left|\int_{0}^{T}\tau_{w}dt\right|}{TAWSS}\right)\;$$

#### 2.5.3. HOLMES

HOLMES is another index that can be considered in cardiovascular simulations [33]. The index presented in Equation (7) combines TAWSS OSI, providing a valuable metric for identifying regions of the vessel that may be prone to abnormal flow-related events [32].

*HOLMES* = *TAWSS* × (0.5 − *OSI*) (7)

#### 2.5.4. ECAP

ECAP represents the level of activity of endothelial cells in response to flow [34]. This index, which is always in the range of 0 to 1.5 Pa^−1^, works in such a way that when its value is above 1.4, the probability of blood clot formation is very high [35]. Equation (8) represents the ECAP [34,36].

*ECAP* = *OSI*/*TAWSS* (8)

### 2.6. Model Assumptions

All in all, the computational model presented in this study is based on the following key assumptions:

- The arterial wall is assumed to be a homogeneous, isotropic, and linear elastic material.
- Blood is modeled as an incompressible, non-Newtonian fluid, with its behavior described by the Carreau–Yasuda model.
- The inlet flow profile is assumed to be a representative physiological waveform based on previously published and validated data for the aortic bifurcation.
- The complex downstream vasculature is represented using 3-element Windkessel models coupled to each outlet, capturing the essential resistance and compliance of the peripheral circulation.
- The stenoses are geometrically modeled as smooth, asymmetric narrowing of the arterial lumen, allowing for a controlled parametric study of stenosis severity.

## 3. Results

### 3.1. Wall Displacement, Pressure, and Velocity

**Figure 3:**
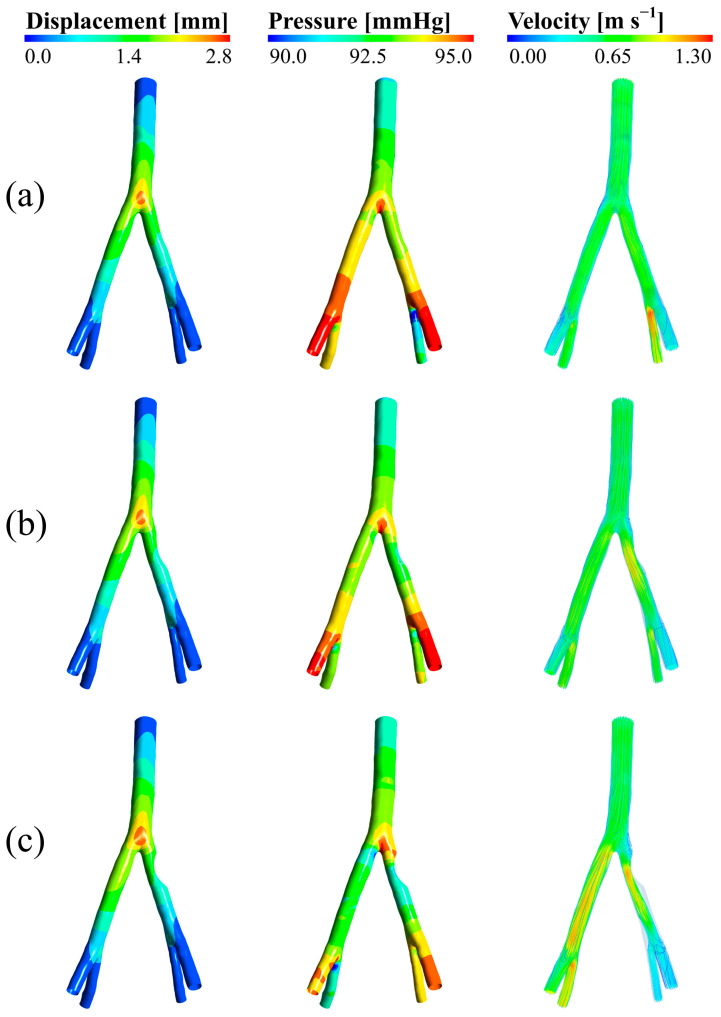
Displacement, pressure, and velocity in (**a**) 25 degrees of stenosis, (**b**) 50 degrees of stenosis, and (**c**) 75 degrees of stenosis.

Figure 3 shows the wall displacement amount in three degrees of involvement of 25, 50, and 75 degrees from the anterior view, respectively, from left to right. According to this figure, the maximum displacement of the wall in all three involvements is related to the iliac bifurcation and its surroundings. It is also quite clear that with increasing the degree of involvement, the displacement values in this part and its surroundings increase. The maximum displacement in these three cases is 2.850 mm, which can be a reasonable and logical value for the iliac artery.

Pressure contours on the left and the velocity streamlines on the right at peak systole for the three aforementioned degrees of stenosis from the anterior view are shown in Figure 3. The results illustrate that the pressure at the iliac bifurcation and in the right and left external iliac branches was high in all three degrees of stenosis. However, the pressure in the right and left internal iliac branches was lower. Furthermore, the pressure on the side with the stenosis was higher than the pressure on the side without the stenosis.

As illustrated in Figure 3, the velocity of blood flow in the stenotic region increased with increasing degree of stenosis. This is because the stenosis causes the blood to flow through a smaller area, which increases the velocity. In terms of velocity streamlines, the noticeable point is the increase in velocity in the stenotic region with increasing degree of stenosis; so much so that in the 75-degree stenosis, even in the left external and internal iliac branches that do not have stenosis, high velocity values can be clearly observed.

### 3.2. TAWSS

**Figure 4:**
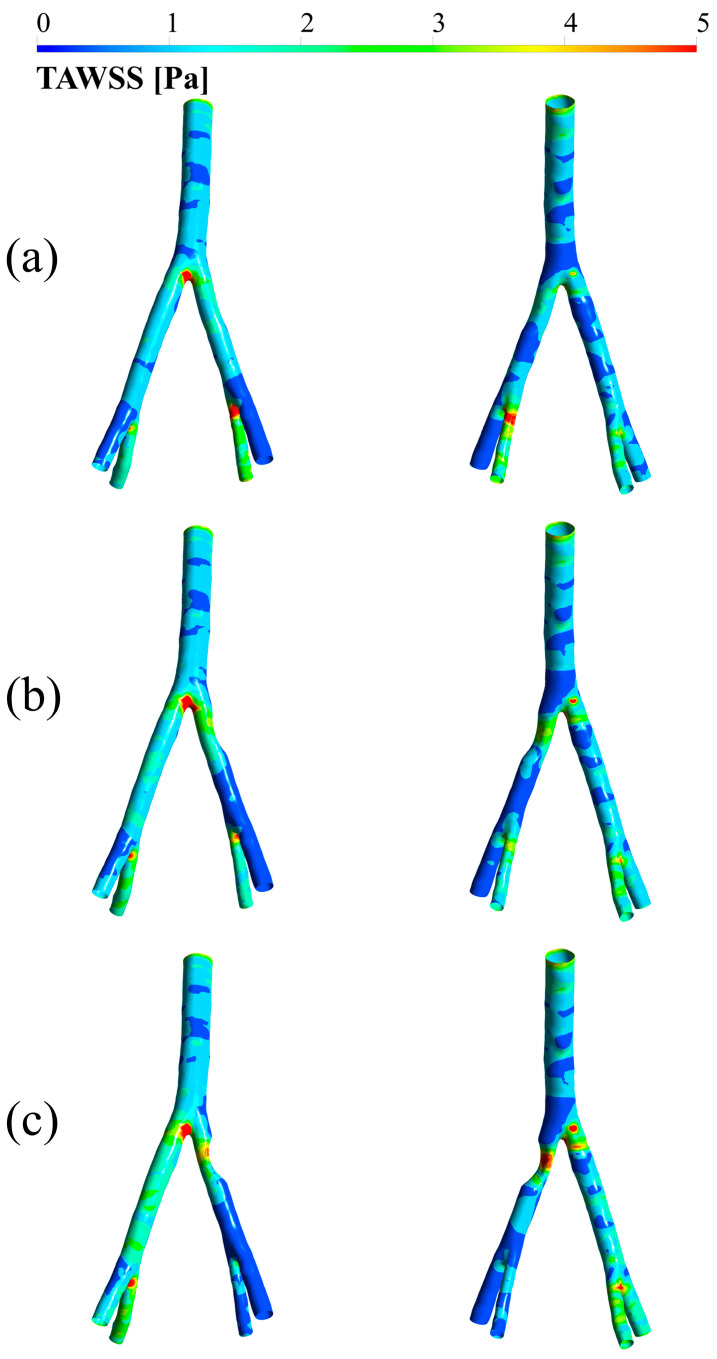
TAWSS in (**a**) 25 degrees of stenosis, (**b**) 50 degrees of stenosis, and (**c**) 75 degrees of stenosis. In each pair, the case on the left represents the anterior view and the case on the right represents the posterior view.

Figure 4 demonstrates TAWSS values in three degrees of stenosis (25, 50, and 75 degrees) and in two views (anterior and posterior). In each pair of images, the left image is from the anterior view, and the right image is from the posterior view. As can be seen, the TAWSS value in all three stenoses occurs at the bifurcation zone. Also, TAWSS values are higher on the side with the stenosis. It can be seen that with an increase in the degree of stenosis, the TAWSS value at the stenosis site increases, which is clearly visible in the 75-degree stenosis. For instance, in the 75% stenosis case, the peak TAWSS within the stenosis reached approximately 4.8 Pa, while the average TAWSS in the region immediately downstream dropped to below 0.4 Pa, a decrease of over 90% from the peak value. This effect was less pronounced but still present in the 25% and 50% cases.

Another point is the significant decrease in TAWSS immediately after stenosis. Higher degrees of stenosis lead to a more severe decrease in this matter. This decrease also occurs with more intensity in the left external and internal iliac branches.

### 3.3. OSI

**Figure 5:**
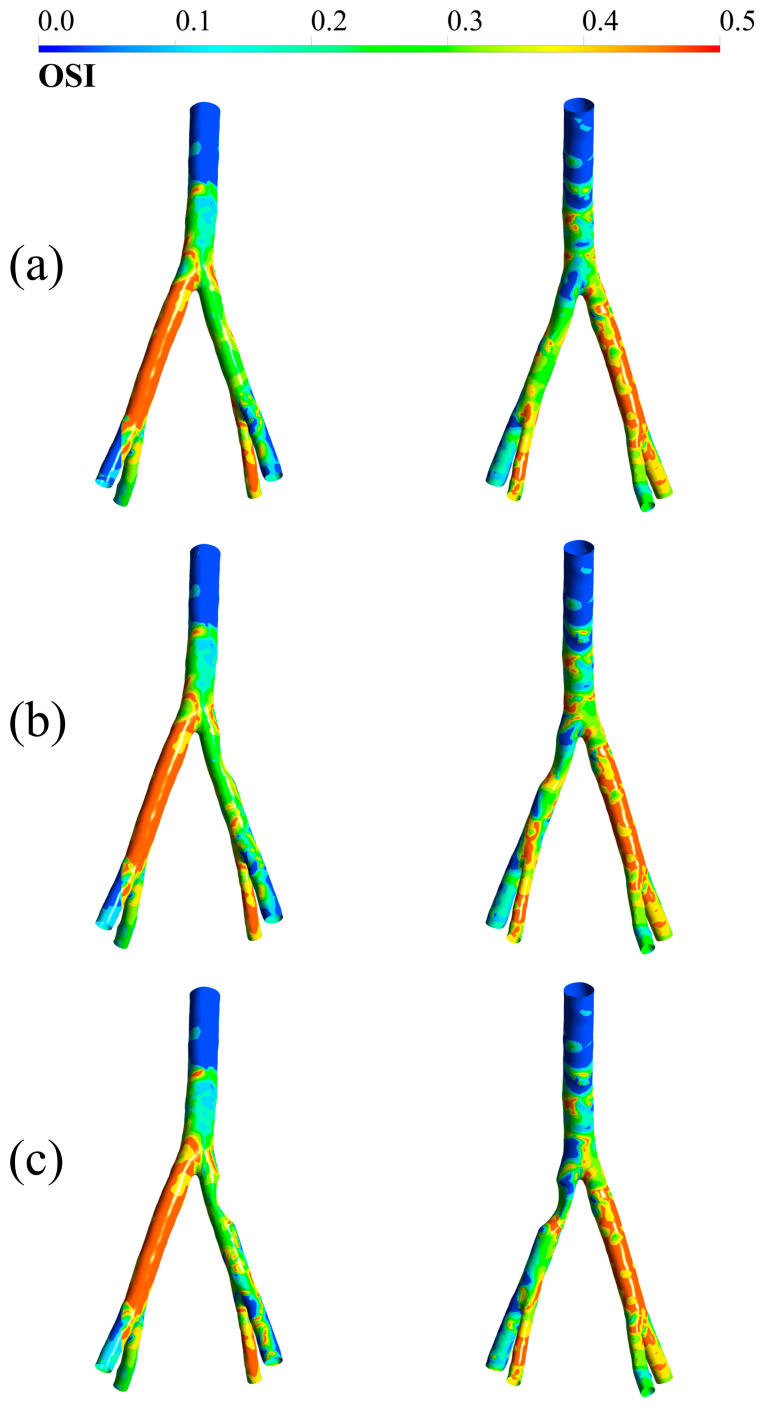
OSI in (**a**) 25 degrees of stenosis, (**b**) 50 degrees of stenosis, and (**c**) 75 degrees of stenosis. In each pair, the case on the left represents the anterior view and the case on the right represents the posterior view.

OSI for three degrees of stenosis—25, 50, and 75 degrees—is shown in Figure 5. As in Figure 4, in each pair of images, the left image is from the anterior view and the right image is from the posterior view. According to Figure 5, in all three stenoses, the highest OSI is related to the side without stenosis, and after that, the bifurcation region has relatively high OSI values. The right internal iliac artery branch also experiences a relatively high OSI. In the stenosis site and its surroundings, moderate values of this index can be seen. It can be observed that after stenosis, in the left external iliac branch, the lower the distance from the stenosis site, the lower the values of the OSI.

### 3.4. HOLMES

**Figure 6:**
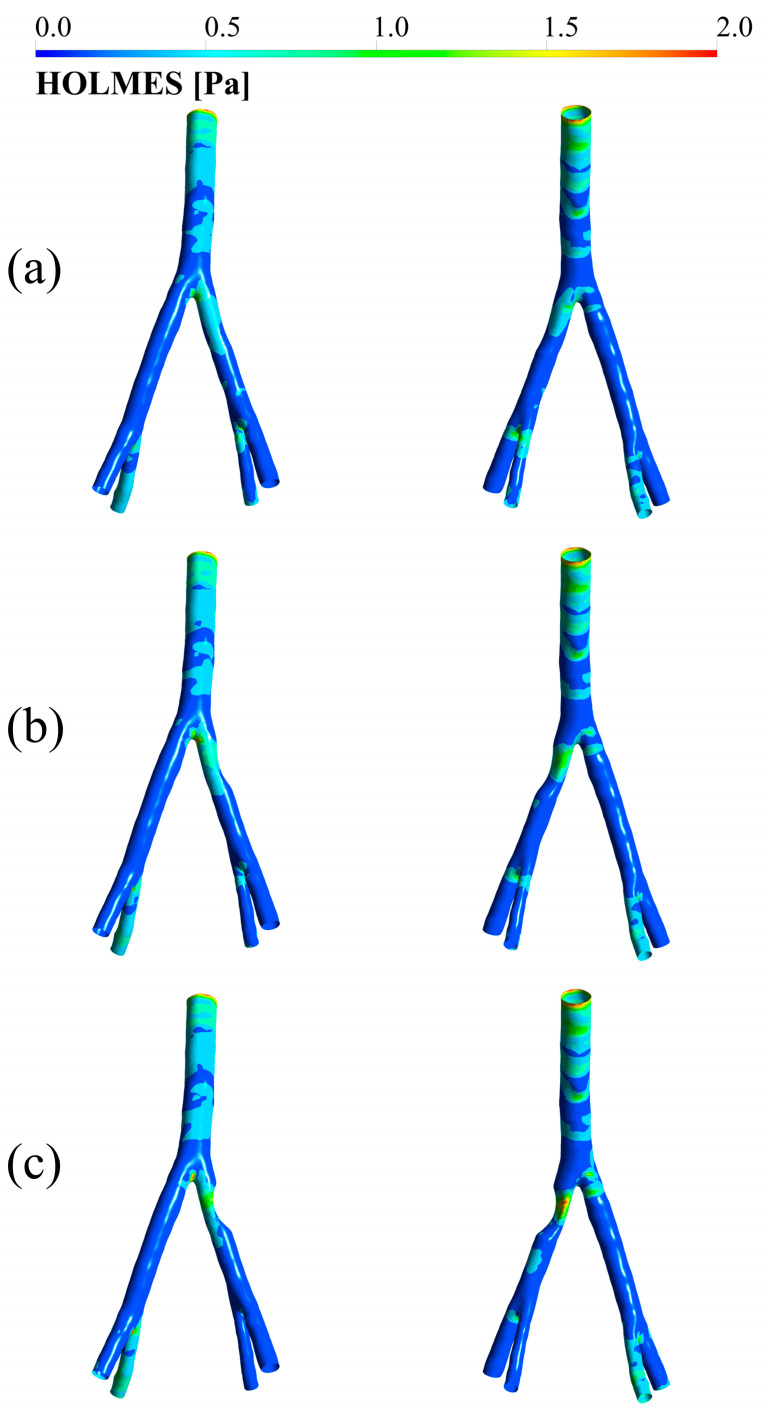
HOLMES index in (**a**) 25 degrees of stenosis, (**b**) 50 degrees of stenosis, and (**c**) 75 degrees of stenosis. In each pair, the case on the left represents the anterior view and the case on the right represents the posterior view.

Figure 6 displays the HOLMES index for the three degrees of stenosis. As shown in the figures, each pair of images displays the anterior view on the left and the posterior view on the right. Figure 6 illustrates that the highest HOLMES index values occur at the stenosis site, with a peak observed at 75% stenosis. Additionally, very low HOLMES values are seen in the iliac branches on both sides, particularly downstream of the narrowed region.

### 3.5. ECAP

**Figure 7:**
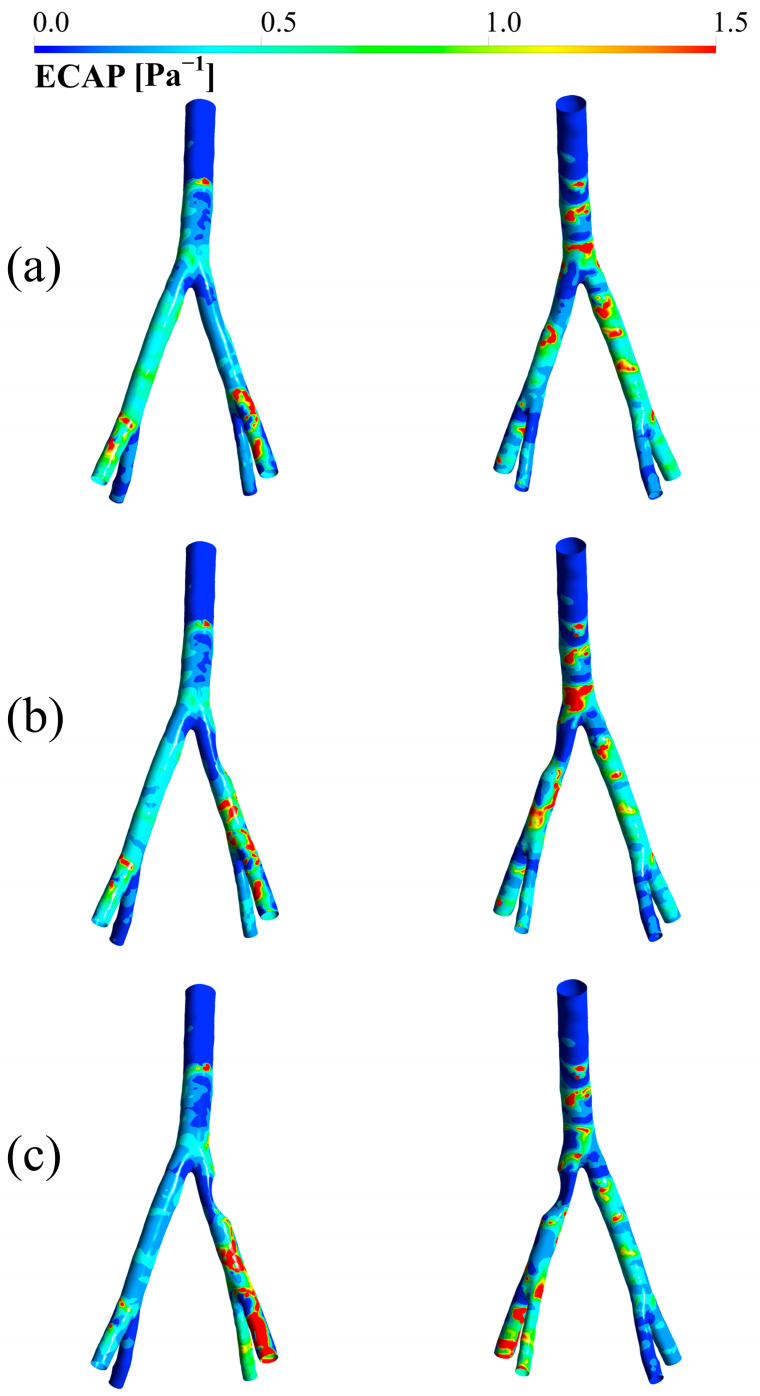
ECAP in (**a**) 25 degrees of stenosis, (**b**) 50 degrees of stenosis, and (**c**) 75 degrees of stenosis. In each pair, the case on the left represents the anterior view and the case on the right represents the posterior view.

Another parameter that needs to be investigated is the potential for endothelial cell activation. Since this index is used to determine the possibility of blood clot formation, it is clear that its values increase in the post-stenosis region with increasing degree of stenosis. This is evident in Figure 7.

## 4. Discussion

The common iliac artery is one of the most vital arteries in the cardiovascular system, playing a crucial role in supplying blood to the lower body [37]. In recent years, computer simulations have emerged as powerful, non-invasive tools in biomedical engineering for predicting, diagnosing, and treating various diseases [38]. CFD and FSI serve as the foundation for these simulations within the cardiovascular field [39]. By applying FSI techniques to semi-idealized geometries reconstructed from patient-specific data, this research has yielded valuable insights into key hemodynamic parameters.

The analysis of wall displacement in iliac arteries with varying degrees of stenosis revealed that displacement increases significantly at and around the bifurcation as stenosis severity intensifies. This is attributed to elevated pressure in the pre-stenotic region, caused by flow obstruction and the accumulation of red blood cells behind the stenosis. These factors collectively impose greater mechanical stress on the bifurcation area, leading to increased wall movement. The range of displacement values aligns well with findings reported by Okazaki et al., validating the use of a linear elastic model for the arterial wall. This modeling choice not only maintained accuracy but also helped to reduce computational costs effectively [40].

Peak systolic pressure and velocity contours were also examined. The results indicate that pressure rises with increasing stenosis severity, particularly in the bifurcation and pre-stenotic zones. This phenomenon results from the impeded flow accumulating behind the stenosis and redirecting toward the unobstructed right branch. Additionally, as expected, velocity increases within the stenotic region as the degree of narrowing progresses. This increase occurs due to pressure drop initiation in the narrowed segment; greater stenosis leads to a more pronounced drop in pressure, thus accelerating the blood flow. This velocity surge can contribute to the formation of recirculation zones [41,42]. These observations are consistent with the outcomes reported by Heinen et al. [19].

TAWSS was another critical parameter explored. Results demonstrate that with greater stenosis, the increase in blood flow velocity heightens the tangential shear force exerted on the vessel wall, thereby elevating TAWSS in the affected region. Since more severe stenoses experience higher velocities, the corresponding TAWSS values also increase accordingly. This may further promote recirculation and flow disturbances [42]. The present findings are supported by similar results from Gorski et al. [43]. Moreover, the detection of very low TAWSS values in post-stenotic areas could indicate increased endothelial cell permeability and a higher likelihood of secondary stenosis formation in those regions [34,44].

OSI, another important hemodynamic metric, was also evaluated. High OSI values were identified in both the common iliac and the internal/external iliac bifurcations. These elevated values are linked to the division of blood flow into two branches, altering its path and the direction of the wall-applied shear stresses. Notably, high OSI was also observed in the right external iliac artery, likely a result of left-side stenosis diverting blood flow toward the right after the bifurcation. This deviation introduces fluctuating, multidirectional forces on the non-stenosed right branch, contributing to elevated OSI in that region, while the stenosed side experiences lower OSI. These findings highlight how both bifurcation geometry and the presence of stenosis influence OSI values. The results are in strong agreement with prior work by Gorski et al. [43], reinforcing their credibility. Since regions exhibiting both high OSI and elevated TAWSS may be prone to future stenosis, clinicians should closely monitor such arterial branches [45,46].

An assessment of the HOLMES index further enriches the analysis. Previous research has suggested that low HOLMES values may indicate abnormal or disturbed flow patterns [25,33]. In this investigation, particularly low HOLMES values were found downstream of stenotic regions, implying that the pressure drop post-stenosis could pose risks even at a distance from the obstruction. This raises concerns that these areas might develop additional stenoses. Consequently, treatment strategies should consider these distal effects to reduce the likelihood of recurrence.

Finally, the ECAP parameter was examined to understand the impact of stenosis on endothelial function. Values above 1.4 are generally associated with a heightened risk of thrombosis due to increased platelet and blood cell aggregation [35]. The study clearly shows that at 75% stenosis, ECAP significantly rises in post-stenotic regions, signaling a potential threat for clot formation in these areas. These results underscore the importance of assessing the entire hemodynamic landscape of a diseased artery rather than focusing solely on the degree of narrowing. For clinicians, this implies that treatment strategies, such as stenting, should aim to restore normal flow patterns both within and downstream of the treated area to mitigate the risk of future thrombotic events or restenosis [45,46]. The computational parameters analyzed here, particularly ECAP and HOLMES, could serve as valuable non-invasive biomarkers for risk stratification and treatment planning in patients with iliac artery disease.

While this study provides valuable insights, its limitations must be acknowledged. First, the use of a single patient-specific geometry, which was then idealized, limits the generalizability of our findings. Future work should incorporate a larger cohort of patient-specific models to validate these results. Second, the arterial wall was modeled as a linear elastic material. While this is a common simplification that reduces computational cost and is a widely accepted approach for studies focusing on blood flow patterns, arterial tissue is known to exhibit hyperelastic and anisotropic properties [29]. Although the overall hemodynamic trends are not expected to change dramatically, the use of a more advanced constitutive model could provide a more accurate representation of wall mechanics, especially under the large deformations seen in the 75% stenosis case. Finally, our model does not account for factors such as stenosis eccentricity, plaque composition, or the presence of arterial branches other than the main bifurcation, which could further influence local hemodynamics. These factors were omitted to maintain computational tractability and focus on isolating the impact of stenosis severity under controlled conditions. Including such anatomical and biochemical complexities would significantly increase modeling complexity, input uncertainty, and computational cost, and may obscure the direct relationship between stenosis degree and hemodynamic parameters. We consider this foundational study an essential step towards more comprehensive future works that will systematically incorporate these additional real-world factors.

## 5. Conclusions

In the present study, using FSI simulation, the effects of different stenosis degrees in the iliac artery on the hemodynamic parameters of blood flow in semi-ideal geometries were investigated. The findings show a significant impact of stenosis on hemodynamic parameters. The FSI simulations carried out on semi-idealized iliac artery models with 25%, 50%, and 75% stenosis demonstrated a clear, progressive alteration of key hemodynamic metrics. Peak wall displacement at the bifurcation rose from about 1.7 mm in the 25% case to 2.1 mm at 50%, reaching 2.85 mm at 75% stenosis, reflecting a nearly 70% increase in wall motion as narrowing intensified. TAWSS similarly escalated within the stenotic throat, more than doubling between the mild and severe scenarios, while immediately downstream, it fell by over 60%, identifying regions prone to endothelial dysfunction. OSI peaked at 0.45 in non-stenosed branches under the 75% case, indicating highly multidirectional flow, and the HOLMES index dropped below 0.3 Pa in post-stenotic zones, signaling disturbed, low-shear environments. Finally, ECAP exceeded the thrombosis risk threshold of 1.4 Pa^−1^ in the post-stenotic region at 75% narrowing, pointing to a markedly increased clot-formation risk. All in all, based on the results of this study, relevant clinicians can conduct further follow-up investigations when treating stenoses in the iliac artery to prevent the occurrence of more stenoses. For instance, these results can help clinicians in endovascular stent placement to observe the effects of stenosis in post-stenotic regions and make decisions based on blood flow after stenosis, which can lead to the treatment of existing stenosis and prevent the development of new stenoses in those areas shortly. Additionally, the investigation of the ECAP index in this study can further assist clinicians in stent placement. Since after stent placement, preventing blood clotting and maintaining blood fluidity is important, and drugs are also prescribed for this purpose, investigating the possibility of blood clot formation in areas near the stenosis can be an important parameter for relevant clinicians. By identifying regions at risk for thrombosis and disturbed flow not only at the stenosis site but also in downstream segments, the results of this study emphasize the importance of comprehensive assessment and targeted follow-up after interventions like stenting. This knowledge can help clinicians optimize treatment strategies to prevent secondary complications and improve long-term outcomes for patients with peripheral arterial disease.

## Figures and Tables

**Table 1:** Parameters for the 3-element Windkessel model at each outlet.

Outlet	Proximal Resistance (R1) (mmHg·s/m^3^)	Distal Resistance (R2) (mmHg·s/m^3^)	Compliance (C) (m^3^/mmHg)
RIIA	2.9982 × 10^4^	1.0002 × 10^−10^	1.5804 × 10^−2^
REIA	2.0584 × 10^4^	1.0002 × 10^−10^	1.3246 × 10^−1^
LIIA	3.7549 × 10^4^	1.0002 × 10^−10^	2.5142 × 10^−2^
LEIA	2.6417 × 10^4^	1.001 × 10^−10^	2.3328 × 10^−1^

**Table 2:** Parameters used for the Carreau–Yasuda model.

$\mu_{0}\;[\mathrm{MPa}\cdot s]$	$\mu_{\infty }\;[\mathrm{MPa}\cdot s]$	a	m	$\lambda_{CY}\;[s]$
22	2.2	0.644	0.392	0.110

## Data Availability

The data presented in this study are available upon reasonable request from the corresponding author.
